# Tumor suppressor protein p53-mediated repression of human mitotic centromere-associated kinesin gene expression is exerted via down-regulation of Sp1 level

**DOI:** 10.1371/journal.pone.0189698

**Published:** 2017-12-15

**Authors:** Do Youn Jun, Ji Young Lee, Hae Sun Park, Yun Han Lee, Young Ho Kim

**Affiliations:** 1 Laboratory of Immunobiology, School of Life Sciences and Biotechnology, College of Natural Sciences, Kyungpook National University, Daegu, Korea; 2 Department of Molecular Medicine, Keimyung University School of Medicine, Daegu, Korea; Virginia Commonwealth University, UNITED STATES

## Abstract

The repressive role of p53 on the human mitotic centromere-associated kinesin (*MCAK*) core promoter from ‒266 to +54, relative to the transcription start site, has been determined. The MCAK mRNA and protein levels were 2.1- and 3.0-fold higher, respectively, in HCT116 (p53^‒/‒^) than in HCT116 (p53^+/+^) cells. Enforced down-regulation of p53 levels either in HCT116 (p53^+/+^) cells by p53 RNAi treatment or in MCF-7 cells using shRNA for p53 (shp53) resulted in a remarkable increase in the MCAK protein level. Site-directed mutagenesis and ChIP analyses showed that p53-mediated repression of the *MCAK* core promoter activity was not directly exerted by p53-binding to putative p53-response elements (p53-RE1 at −173/−166 and p53-RE2 at −245/−238), but indirectly by attenuating Sp1 binding to GC-motifs (GC1 at −93/−84 and GC2 at −119/−110). Treatment of HEK-293 cells bearing the *MCAK* core promoter-reporter (pGL2-320-Luc) with mithramycin A, which down-regulates Sp1 gene expression, reduced the promoter activity as well as endogenous MCAK levels. Exposure of HCT116 (p53^+/+^) cells to nutlin-3a, a validated activator of p53, caused a simultaneous reduction in Sp1 and MCAK protein levels, but not in HCT116 (p53^−/−^) cells. In contrast to wild-type (wt)-p53, tumor-derived p53 mutants (p53^V143A^, p53^R248W^, and p53^R273H^) failed to repress the Sp1-dependent activation of the *MCAK* promoter and to down-regulate endogenous levels of Sp1 and MCAK proteins. Collectively, these findings demonstrate that p53 can repress *MCAK* promoter activity indirectly via down-regulation of Sp1 expression level, and suggest that MCAK elevation in human tumor cells might be due to p53 mutation.

## Introduction

MCAK has been the best-characterized member of the kinesin-13 family in terms of subcellular localization and microtubule-depolymerizing activity regulation. During cell cycle progression, MCAK is dominantly located at the centrosomes, centromere and kinetochores, spindle poles, and midbody [1–3]. The activity of MCAK appears to be regulated not only via association with cofactors such as Inner Centromere KinI Stimulator (ICIS) [4], hSgo2, EB1, and TIP150, but also via alteration of the phosphorylation status that is mediated by mitotic kinases including Cdk1 [5], Aurora B [6–8], Aurora A [9], and Polo-like kinase 1 [10, 11].

Chromosomal aneuploidy, an abnormality in the number of chromosomes, is considered one of the most common features of cancer. Defects in segregation fidelity during mitosis can cause chromosome instability (CIN), which is defined as persistent mis-segregation of whole chromosomes during cell division [12]. Several studies have shown that aneuploidy and CIN result in increased metastasis and resistance to chemotherapeutics, which is relevant to high recurrence and mortality rates [13]. Such aneuploidy and CIN occur when there is impairment of the coupling between the microtubules of the mitotic spindle and the chromosomes [14, 15]. Consequently, since microtubule dynamics is crucial for proper binding of chromosomes to mitotic spindles as well as segregation of the chromosomes to the daughter cells during mitotic cell division, it is likely that any cellular changes causing defects in the control of microtubule dynamics could lead to CIN [16]. A remarkably enhanced level of human MCAK gene expression has been indicated in several human cancers, including breast cancer [17, 18], gastric cancer [19], and colorectal cancer [20]. Because overexpression of MCAK can increase the frequency of monopolar spindles [21] and multipolar spindles [22], it has been suggested that elevated MCAK levels might contribute to either the gain or loss of chromosomes in daughter cells, leading to tumorigenesis.

In a previous study, we cloned the human *MCAK* promoter region from −1097 to +54 (pGL2-1151-Luc) relative to the transcription start site in order to elucidate the transcriptional regulation of the human MCAK gene [23]. Mutational and chromatin immunoprecipitation (ChIP) analyses of the *MCAK* core promoter region (−266/+54) in HEK-293 cells showed the GC-motifs (GC1 at −93/−84 and GC2 at −119/−110) are crucial for promoter activation. Additionally, the analysis with the serial deletion constructs of the *MCAK* promoter suggested that the promoter region (–476/–267) possessing a putative p53-response element (p53-RE at −421/-401; CACCGCGCCCGGCCAAGTCTC) might be associated with promoter repression. The putative p53-RE appeared to resemble the consensus p53 binding sequence, consisting of two repeats of a 10-bp motif, 5′-PuPuPuC(A/T)(T/A)GPyPyPy-3′, separated by a 0- to13-bp spacer [24]. Previously, Shimo *et al*. also demonstrated that ectopic overexpression of p53 caused a significant repression of MCAK gene expression in human breast cancer cells [18]. However, the molecular regulatory mechanisms underlying the p53-mediated negative regulation of MCAK gene expression as well as the tumor-associated elevation in MCAK level still remain unclear.

Recently, we found two additional putative p53-response elements (p53-RE1 at −173/−166 and p53-RE2 at −245/−238) within the human *MCAK* core promoter region (−266/+54), which was previously shown to contain two Sp1-binding motifs (GC1 at −93/−84 and GC2 at −119/−110) for positive regulation of the promoter activity [23]. In the present study, to examine the mechanisms responsible for p53-mediated repression of MCAK gene expression and upregulation of MCAK level in tumor cells, we investigated whether the human *MCAK* core promoter activity is repressed through p53 binding to putative p53-RE1 and p53-RE2. Additionally, we investigated whether p53-dependent down-regulation of Sp1 level contributes to p53-mediated repression of the *MCAK* promoter activity, because Sp1 gene expression was reported to be negatively regulated by p53 [25]. The results demonstrate that p53-mediated repression of the *MCAK* promoter activity is not exerted by p53 binding to its response elements (p53-REs), but by p53-dependent down-regulation of Sp1 which is crucial for transcriptional activation of the *MCAK* core promoter. The results also suggest that tumor-associated elevation of MCAK levels might result from p53 mutation.

## Materials and methods

### Enzymes, reagents, antibodies and cells

All restriction and modifying enzymes, reporter gene vectors (pGL2-basic, pGL2-promoter, pSV-β-gal) were purchased from Promega (Madison, WI, USA). An ECL Western Blotting Kit was purchased from GE Healthcare Life Sciences (Pittsburgh, PA, USA) and a ChIP Assay Kit was purchased from EMD Millipore (Billerica, MA, USA). Mithramycin A and normal mouse IgG were`purchased from Sigma-Aldrich (St. Louis, MO, USA). A rabbit polyclonal anti-human MCAK antibody was raised against the N-terminal region (187 amino acids) of human MCAK [26]. Anti-CDK4, anti-p53, anti-p21^WAF1/Cip1^, anti-Sp1, and anti-β-actin antibodies were purchased from Santa Cruz Biotechnology (Dallas, TX, USA). Anti-p-p53 (Ser-15) and anti-poly (ADP-ribose) polymerase (PARP) antibodies were obtained from Cell Signaling Technology (Beverly, MA, USA), and anti-p-Sp1(Thr-453) antibody was obtained from Abcam (Cambridge, MA, USA). Human colorectal adenocarcinoma cell lines HCT116 (p53^+/+^) and p53-null HCT116 (p53^−/−^) were kindly provided by Dr. B. Vogelstein (Johns Hopkins University, Baltimore, MD, USA). Human embryonic kidney cell line HEK-293, human cervical cancer cell line HeLa, and human colon adenocarcinoma cell line SW480 were purchased from ATCC (Manassas, VA, USA). Human breast adenocarcinoma cell line stably transfected with shRNA control vector (MCF-control) and MCF7 cells stably transfected with shp53 expression vector (MCF7-shp53) were kindly provided by Chris R. Harris (University of Medicine and Dentistry of New Jersey, New Brunswick, NJ, USA). Cells were grown in Dulbecco's modified Eagle's medium (Invitrogen, Life Technology, Grand Island, NY, USA) supplemented with 10% fetal calf serum and 100 μg/mL of gentamycin.

### Plasmids

Generation of *MCAK* promoter-reporter plasmids containing DNA fragments (−476/+54) or (−266/+54) of the 5'-regulatory region was previously described [23]. The expression plasmids (pCMV-Neo-wt-p53, pCMV-Neo-mutant p53^V143A^, pCMV-Neo-mutant p53^R248W^, and pCMV-Neo-mutant p53^R273H^) were provided by Dr. B. Vogelstein (Johns Hopkins University). The mdm2-luciferase construct as a p53-response positive reporter control was provided by Dr. M. Oren (Weizmann Institute of Science, Rehovot, Israel). The pG5-5x(GC)-Luc construct containing five tandem Sp1-binding sites was provided by Dr. Man-Wook Hur [27]. The p53-RE and GC-motif mutant constructs were prepared as described previously [23]. The expression vector plasmids pCAGGS-Sp1 and pCAGGS-wt-p53 were prepared using the pCAGGS expression vector [28].

### Transient transfection and luciferase assay

HEK-293 or HCT116 cells were transfected by using Lipofectamine 2000 reagent (Invitrogen). The pSV-β-galactosidase control vector was used to normalize the transfection efficiency. After transfection for 36−48 h, luciferase activity was measured using the Luciferase Reporter Assay System (Promega) and β-galactosidase activity was determined using the Galacto-Light Plus Kit (Tropix Inc., Bedford, MA, USA).

### RNA isolation, cDNA synthesis, and quantitative real-time RT-PCR

Total RNA (10 μg) was transcribed using Superscript II (Invitrogen) and random hexamer primers, according to the manufacturer’s instructions. PCR amplification was performed using AccuPrime Tag DNA polymerase high fidelity (Invitrogen) and specific primers. The following primers were used for PCR: MCAK-forward, 5'-ACGGAATTCCTGAGATATGCAGAC-3'; MCAK-reverse, 5'-AATGGAT-CCTCAATGCTTGGCTTGCTG-3'; p53-forward, 5'-ACCACCATCCACTACAACTAC-3'; p53-reverse, 5'-AAGGATCCTCAGTCTGAGTCAGGCC-3'; p21-forward, 5'-GTTCCTTGTGGAGCCG-GAGC-3'; p21-reverse, 5'-GGTACAAGACAGTGACAGGTC-3'; glyceraldehyde 3-phosphate dehydrogenase (GAPDH)-forward, 5'-CCACTGGCGTCTTCACCAC-3'; GAPDH-reverse 5'-CCTG-CTTCACCACCTTCTTG-3'. The PCR products were electrophoresed using 1.5% agarose gels, and visualized after ethidium bromide staining under UV light.

### Chromatin immunoprecipitation (ChIP) assay

Formaldehyde cross-linking and ChIP assay were conducted using a ChIP Assay Kit as described previously [23]. Briefly, formaldehyde cross-linked chromatin was prepared from HCT116 (p53^-/-^) cells transiently transfected with wild-type p53-expression vector (pCMV-Neo/p53) or empty-vector (pCMV-Neo), and was immunoprecipitated with anti-Sp1 antibody. Normal mouse IgG was also included as a negative control. PCR-amplification of the *MCAK* 320-bp promoter region (−266/+54) was performed using the *Sac*I-forward primer (5′-AGGAGCTCGGTGACCGAGGAC-AAT-3′) and the *Bgl*II-reverse primer-2 (5′-AGAGATCTCGGAGAGTCAGCAAGGAAGAG-3′).

### Stealth RNA interference (RNAi) expression

To reduce the level of p53 protein in HCT116 (p53^+/+^) cells, Stealth RNAi for p53 (#1: 5′-CCGG-ACGAUAUUGAACAAUGGUUCA-3′, #2: 5′-AGUACGUGCAAGUCACAGACUUGGC-3′) was transfected into the cells at a concentration of 200 nM by using Lipofectamine 2000. Cell lysates were prepared 48 h after transfection. Synthetic Stealth RNAi duplex for p53 and scrambled Stealth RNAi duplex (Cat. #12395–100) for a negative control were designed and supplied by Invitrogen.

### Preparation of cell lysate and western blot analysis

Preparation of cell lysates and western blot analysis were performed as described previously [23]. An equivalent amount of cell lysate (20 μg) was electrophoresed on 4~12% NuPAGE gradient gels (Invitrogen Corporation/Novex, Carlsbad, CA, USA) with MOPS buffer and then electrotransferred to a nylon membrane. Detection of individual proteins was performed using an ECL Western Blotting Kit, according to the manufacturer's instructions. Densitometry was performed by using ImageQuant TL software (GE Healthcare Life Sciences). Arbitrary densitometric units of the protein of interest were normalized to those of β-actin.

### Statistical analysis

Unless otherwise indicated, each result in this paper is representative of at least three separate experiments. Statistical analyses were performed using the Students t-test to evaluate the significance of differences between two groups and one-way ANOVA for between three or more than three groups. In all graphs, * indicates *p* < 0.05 between the untreated and treated cells. All data are expressed as the mean ± standard deviation (SD, for each group n≥3). One-way ANOVA followed by Dunnett’s multiple comparison test was also used for statistical analysis using the SPSS Statistics version 23 (IBM, Armonk, NY, USA).

## Results and discussion

### Enhanced expression of MCAK by p53-deficiency

**Fig 1:**
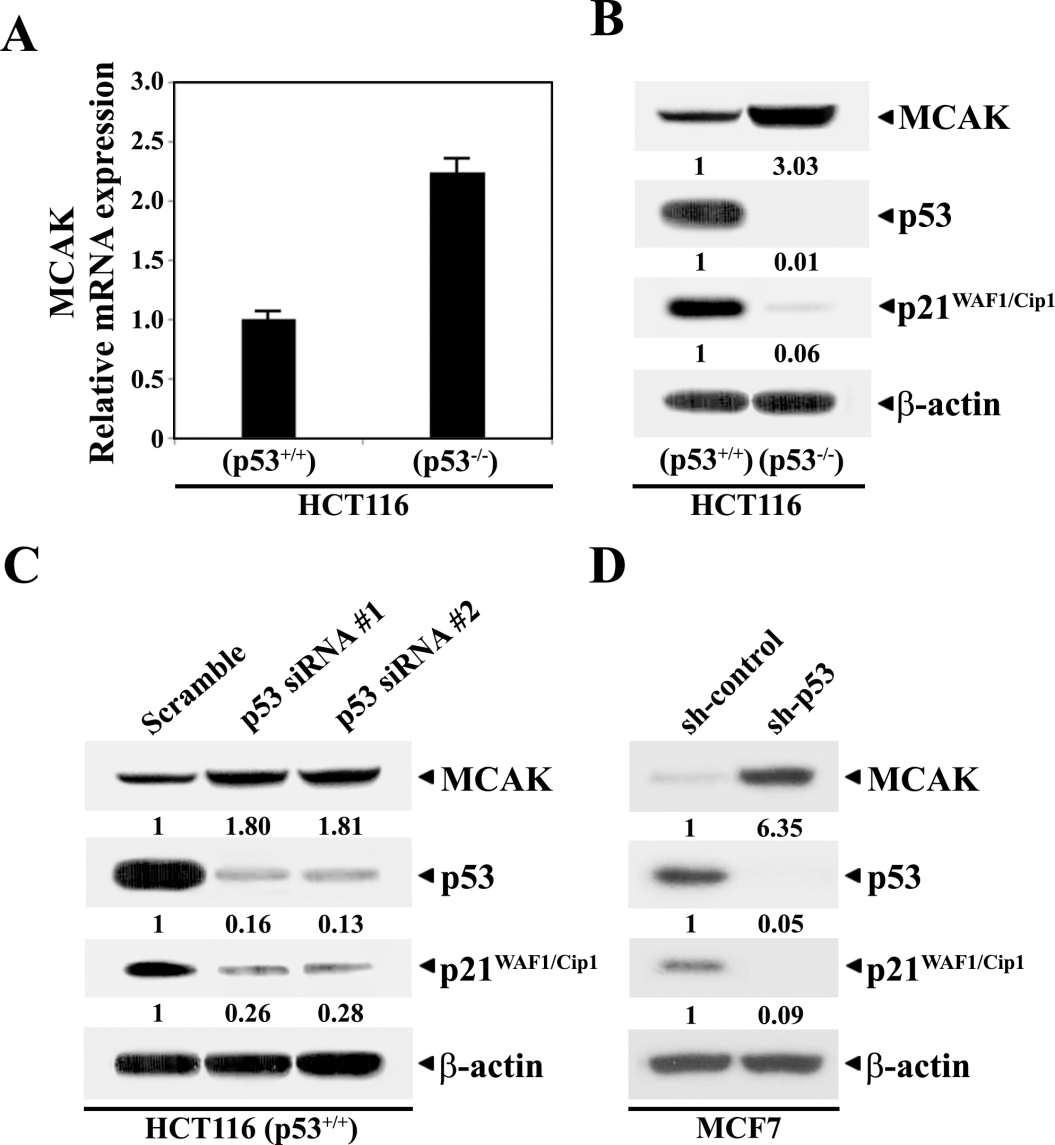
**Semi-quantitative RT-PCR analysis of mRNAs specific for MCAK and GAPDH (A), western analysis of protein levels of MCAK, p53, p21**^**WAF1/Cip1**^**, and β-actin in continuously growing HCT116 (p53**^**+/+**^**) and HCT116 (p53**^−**/**−^**) cells (B), and western blot analysis of individual protein levels in HCT116 (p53**^**+/+**^**) cells following treatment with the control scramble RNAi or Stealth RNAi for p53 (C), and in MCF7 cells stably transfected with control shRNA vector or shp53 vector (D).** RT-PCR was performed using gene-specific primers as described in Materials and methods. To ensure that the same amount of RNA was being used, the total RNA concentration was normalized to that of GAPDH as the message of a housekeeping gene. HCT116 (p53^+/+^) cells were treated with each RNAi for 48 h. Western blot analysis was performed using ECL Western Blotting Kit as described in Section 2. A representative study is shown and two additional experiments yielded similar results.

To examine the involvement of p53 as a transcriptional repressor in the MCAK gene expression, the MCAK-specific mRNA and protein levels were compared between human colorectal adenocarcinoma HCT116 (p53^+/+^) and HCT116 (p53^−/−^) cells. The level of p21^WAF1/Cip1^, the expression of which is known to be activated by p53 [29], was also investigated to verify the specificity of the p53 effect on MCAK gene expression. The expression levels of MCAK mRNA and protein appeared to be 2.1- and 2.3-fold higher in p53-null HCT116 (p53^−/−^) cells, respectively, in comparison to those in HCT116 (p53^+/+^) cells (Fig 1A and 1B). Additionally, the level of p21^WAF1/Cip1^ protein became barely detectable in the HCT116 (p53^−/−^) cells. These results indicate that the expression of the MCAK gene could be repressed at the transcriptional level by the function of p53.

To further examine the repressive role of p53 in the expression of MCAK gene, we investigated, by western blot analysis, whether knock-down of p53 expression by employing Stealth RNAi specific for p53 could enhance the MCAK protein level by ~1.8-fold in HCT116 (p53^+/+^). Although treatment with the control scramble RNAi did not influence p53 protein level in the cells, treatment with two different kinds of p53-specific RNAi could reduce p53 protein levels by 0.16- and 0.13-fold (Fig 1C). While the p53 level declined, the MCAK level was enhanced and the p21^WAF1/Cip1^ level was markedly down-regulated. The p53-dependent decrease in the MCAK level was also observed when the MCAK level was compared between human breast adenocarcinoma MCF-control cells and MCF7-shp53 cells (Fig 1D). These results indicate that the p53-mediated negative regulation of MCAK gene expression occurs commonly in colon cancer HCT1116 and breast cancer MCF7 cells.

### p53-mediated repression of the MCAK proximal promoter activity

**Fig 2:**
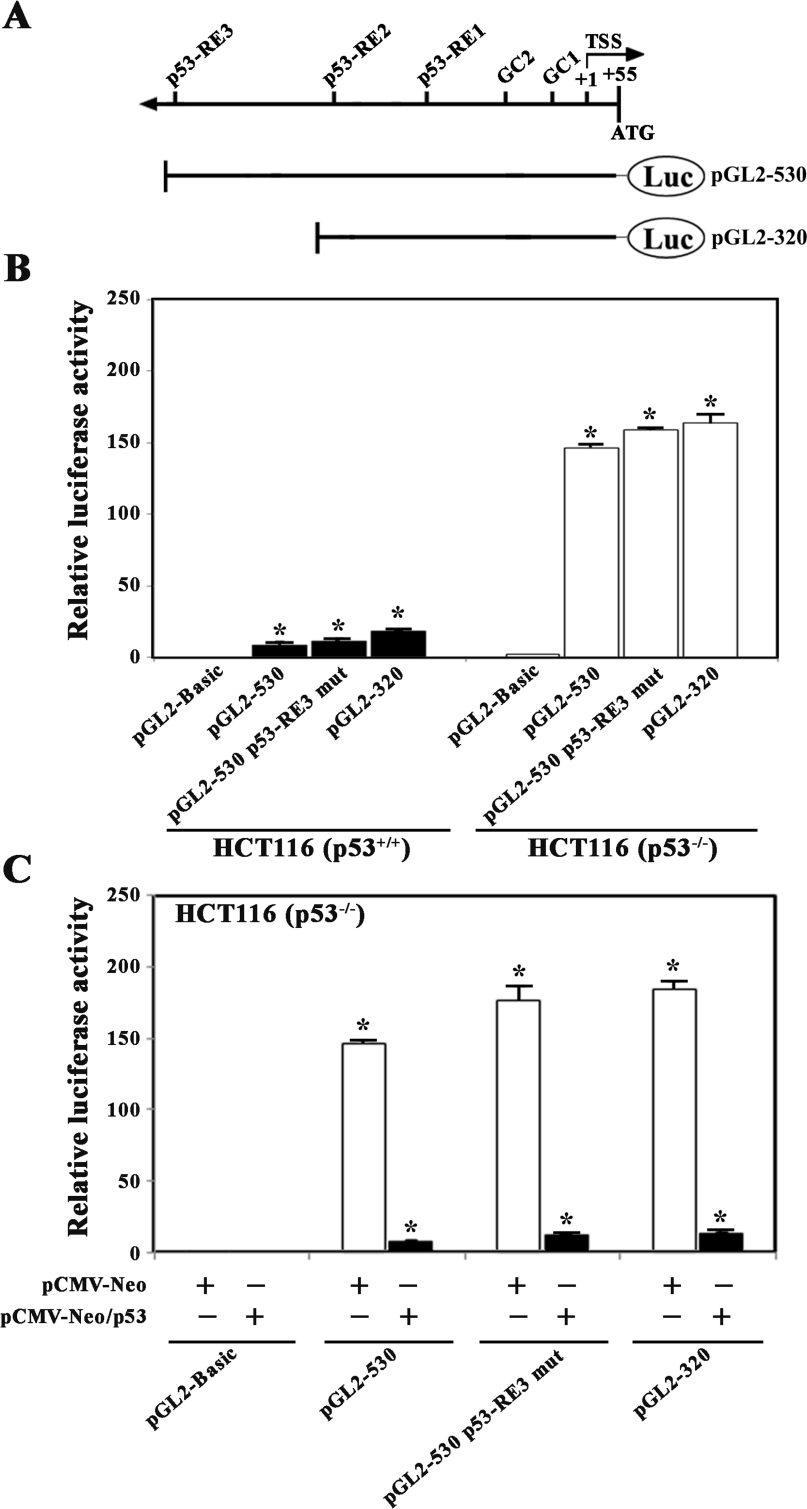
**Diagrammatic representation of the transcription factor binding sites in the *MCAK* promoter-reporter constructs (A), comparison of the promoter activities among pGL2-basic, pGL2-530, pGL2-530/mutated p53-motif, and pGL2-320 constructs in HCT116 (p53**^**+/+**^**) cells (B), and effect of ectopically expressed p53 on the promoter activities of pGL2-530, pGL2-530/mutated p53-motif, and pGL2-320 constructs in p53-deficient HCT116 cells (C).** The cells were transiently transfected or cotransfected with individual the promoter-reporter constructs with wild-type p53-expression vector (pCMV-Neo/p53) or empty-vector (pCMV-Neo). The pSV-β-galactosidase control vector was cotransfected in each experiment to correct for variations in transfection efficiency. The luciferase and β-galactosidase activities were measured as described in Materials and methods. Each value is expressed as the mean ± SD (n = 3). *Statistical significance was defined as *p*<0.05 compared to the control.

To examine whether the human *MCAK* promoter has p53-response element (p53-RE) involved in promoter repression, the proximal promoter sequences (−476/+54) were analyzed using two transcription factor binding prediction programs (http://www.cbil.upenn.edu/cgi-bin/tess/tess and http://motif.genome.jp). As shown in Fig 2A, three putative p53-response elements (designated as p53-RE1 at −173/−166, p53-RE2 at -245/-238, and p53-RE3 at −421/−401) as well as two Sp1-binding motifs (designated as GC1 at −93/−84 and GC2 at −119/−110) were detected in the *MCAK* proximal promoter sequences. To investigate the functional role of the three putative p53-REs in the repression of *MCAK* promoter activity, the promoter activities of pGL2-530-Luc (the reporter construct containing the promoter region from −476 to +54), pGL2-530-Luc with p53-RE3 mutation, and pGL2-320-Luc (the reporter construct containing the promoter region from -266 to +54) were compared in HCT116 (p53^+/+^) and HCT116 (p53^−/−^) cells. As shown in Fig 2B, neither the mutation of p53-RE3 at −421/−401 (CACCGCGCCCGGCCAAGTCTC → CACCGCGCCCGGCaggagCTC) nor the deletion of p53-RE3 produced a remarkable enhancement in the promoter activity in HCT116 (p53^+/+^) cells. The luciferase activities of these reporter constructs (pGL2-530-Luc, pGL2-530-Luc with p53-RE3 mutation, and pGL2-320-Luc) were enhanced ~16.6-, 14.3-, and 9.0-fold in HCT-116 (p53^−/−^) cells, respectively, as compared with the activities in HCT116 (p53^+/+^) cells. However, the enhanced activities of the individual reporter constructs were abrogated when these reporter constructs were cotransfected with wt-p53 expression vector (pCMV-Neo-wt-p53) into HCT116 (p53^−/−^) cells (Fig 2C). These results suggest that p53-RE1 and/or p53-RE2 rather than p53-RE3 might be more critical for p53-mediated repression of the *MCAK* core promoter activity.

**Fig 3:**
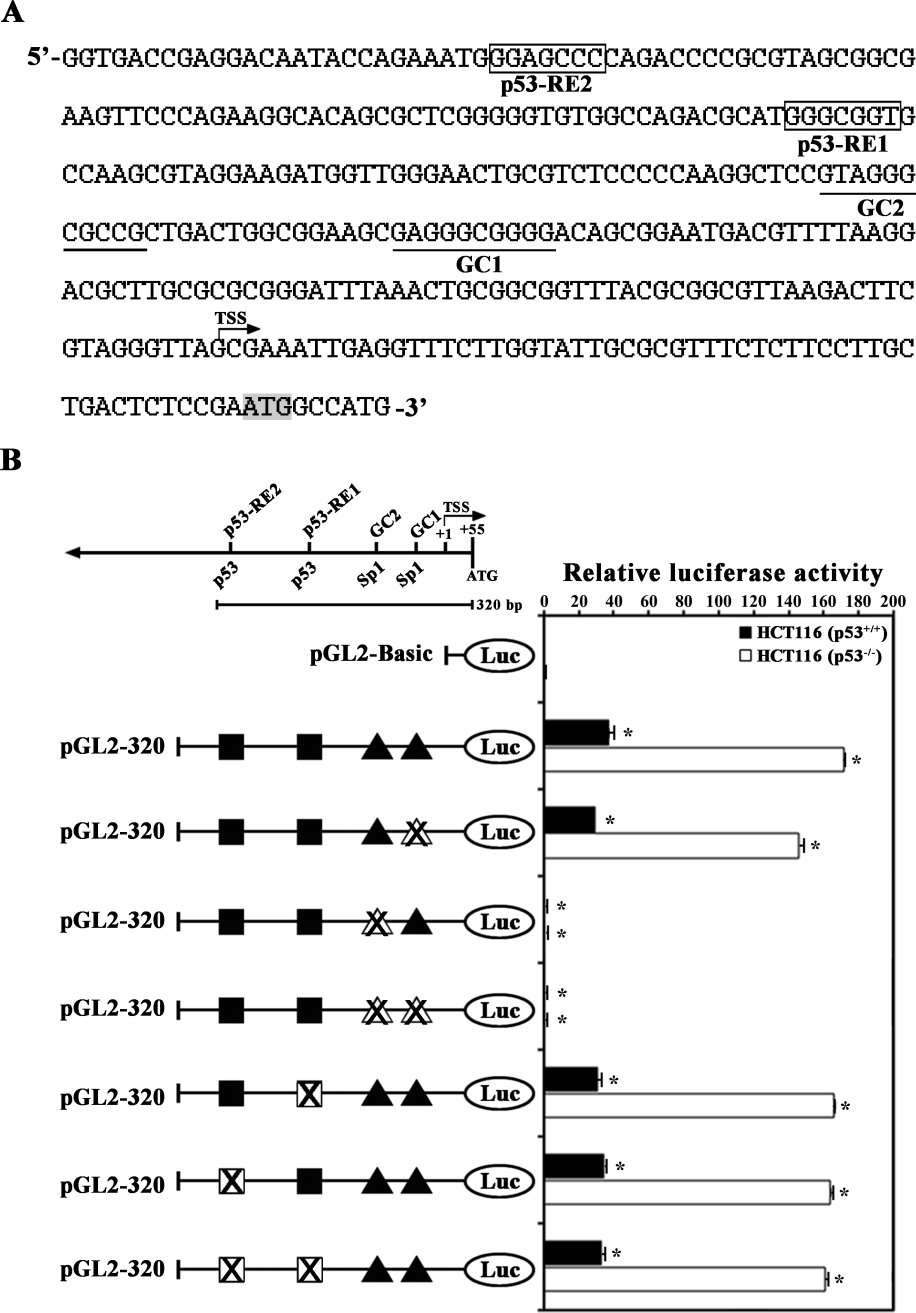
**Nucleotide sequence and putative transcription factor binding sites (A), and mutational analysis of the two p53-REs (p53-RE1 at −173/−166 and p53-RE2 at −245/−238) and two Sp1 binding motifs (GC1 at −93/−84 and GC2 at −119/−110) (B) in the human *MCAK* promoter (pGL2-320-Luc).** The nucleotide sequence of the *MCAK* promoter region from −266 to +54 was analyzed and the positions of the putative transcription factor binding site are underlined, and the transcription start site is designated as +1. The translation start codon ATG is shaded light gray. The individual site-directed mutations were introduced within the pGL2-320-Luc vector. Transient transfection with each mutation construct was performed in HCT116 (p53^+/+^) and HCT116 (p53^−/−^) cells, and then luciferase activity was determined. Each value is expressed as the mean ± SD (n = 3). *Statistical significance was defined as *p*<0.05 compared to the control.

To investigate the involvement of p53-RE1 and p53-RE2 in the p53-mediated repression of the *MCAK* core promoter, mutant constructs of pGL2-320-Luc, possessing site-directed mutations in p53-RE1 at −182/−155 and p53-RE2 at −256/−228 were transfected into HCT116 (p53^+/+^) and HCT116 (p53^−/−^) cells, and then individual promoter activities were analyzed. The nucleotide sequence, putative transcription factor binding sites, and transcription start site (TSS) are shown in the *MCAK* 320-bp promoter region (−266/+54) (Fig 3A). Mutations in p53-RE1 at −182/−155 (CAGACGCAT-GGGCGGTGCCAAGCGTAGG → CAGACGCATGGtttGTGCCAAGCGTAGG) and in p53-RE2 at −256/−228 (ATACCAGAAATGGGAGCCCC-AAACCCCG → ATACCAGAAATGttgaCCCC-AAACCCCG) failed to influence the promoter activity in both HCT116 (p53^+/+^) and HCT116 (p53^−/−^) cells (Fig 3B). At the same time, the individual promoter activities detected in HCT116 (p53^+/+^) cells were reduced to 19−21% of those detected in HCT116 (p53^-/-^) cells. These results demonstrate that there was no contribution from either p53-RE1 or p53-RE2 to p53-mediated repression of the *MCAK* core promoter activity. On the other hand, mutation in the distal GC-motif (GC2) at −119/−110 (GCAGGGCGCC→ GCAGttaGCC) completely abrogated the promoter activity irrespective of the presence of p53, whereas mutation in the proximal GC-motif (GC1) at −93/−84 (GAGGGCGGGG→ GAGttaGGGG) resulted in a decrease in the promoter activity to ~85% and ~77% of the pGL2-320-Luc activity in HCT116 (p53^−/−^) and HCT116 (p53^+/+^) cells, respectively. These results indicate that although both GC-motifs (GC1 and GC2) were required for positive regulation of the *MCAK* promoter in HCT116 cells, the GC2-motif was more critical than GC1 for the *MCAK* promoter activity.

**Fig 4:**
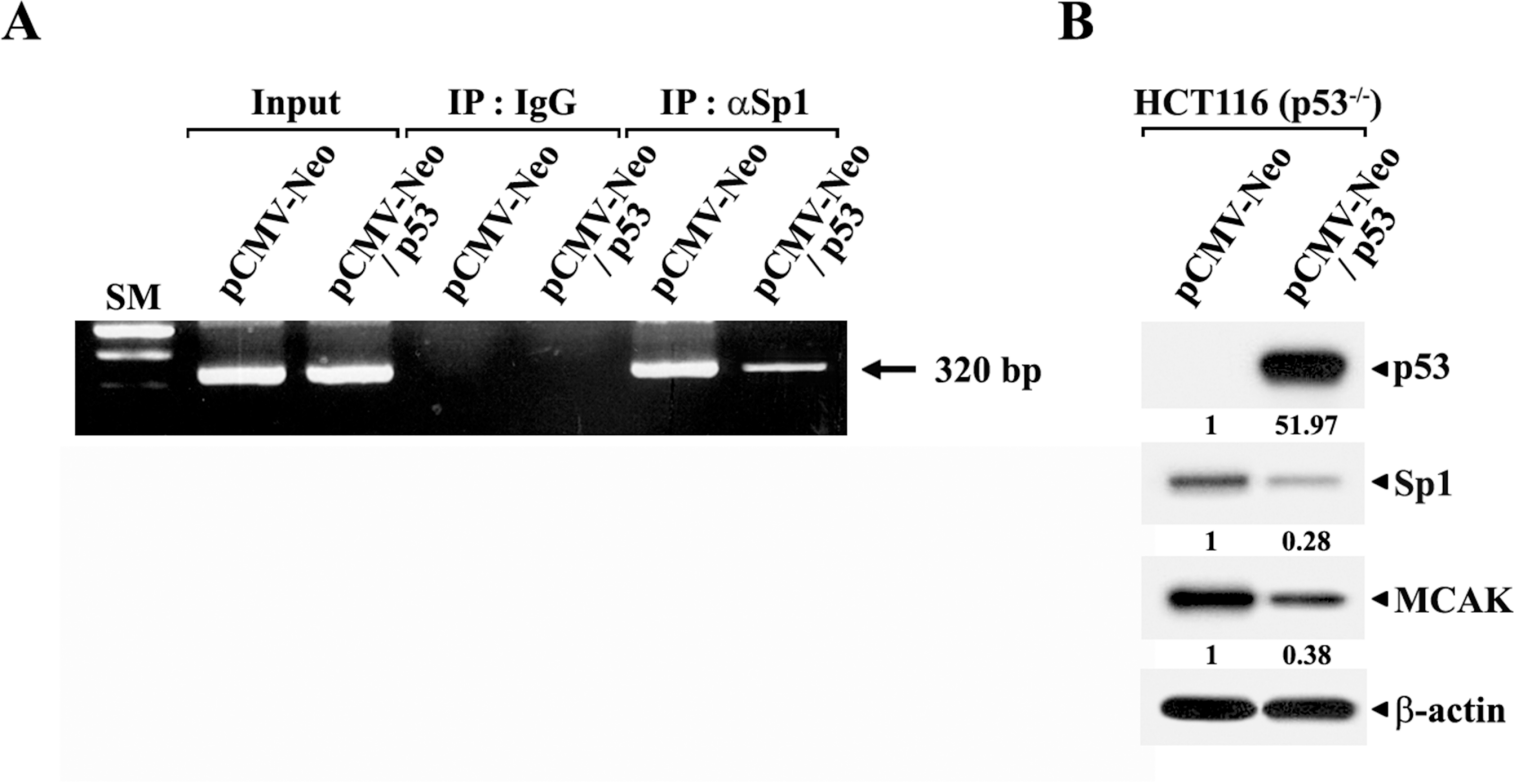
**ChIP analyses to detect the suppressive effect of p53 overexpression on the *in vivo* association of Sp1 with the *MCAK* promoter (A) and western blot analyses of individual protein levels in HCT116 (p53**^−**/**−^**) cells (B). The cells were transiently transfected with wild-type p53-expression vector (pCMV-Neo/p53) or empty-vector (pCMV-Neo).** ChIP assays were performed using a ChIP Assay Kit. Formaldehyde-cross-linked chromatin was prepared from the cells and then immunoprecipitated with anti-Sp1 antibody or normal mouse IgG. The recovered immunoprecipitates were analyzed by PCR with specific primers to amplify the 320-bp *MCAK* promoter region (−266/+54) possessing two p53-REs (p53-RE1 at −173/−166 and p53-RE2 at −245/−238) as well as two GC-motifs (GC1 at −93/−84 and GC2 at −119/−110) using the *Sac*I-forward primer (5'-AGGA-GCTCAGTCAAGTTTCTAATCTG-3') and the *Bgl*II-reverse primer-2 (5’-AGAGATCTCGGAGA-GTCAGCAAGGAAGAG-3’). The 320-bp PCR products were resolved on a 1.5% agarose gel electrophoresis and are indicated by arrows. Western blot analyses were performed as described in Materials and methods. A representative study is shown and two additional experiments yielded similar results.

To further examine the mechanism underlying p53-mediated repression of the Sp1-dependent activation of the *MCAK* core promoter, the effect of p53 on Sp1-binding to the *MCAK* core promoter was investigated by ChIP assay in HCT116 (p53^−/−^) cells following transfection with pCMV-Neo-wt-p53. As shown in Fig 4A, the ectopic expression of wt-p53 in HCT116 (p53^−/−^) resulted in a remarkable decrease in the level of Sp1-binding to GC-motifs located within the *MCAK* core promoter. Western blot analysis also showed that the expression levels of endogenous Sp1 and MCAK proteins were reduced 0.28- and 0.38-fold by the ectopic expression of wt-p53 (Fig 4B). Although the Sp1 family of transcription factors has been reported to include several Sp1-related proteins, such as Sp2, Sp3, and Sp4, which share the DNA-binding zinc-finger domain [30], and that the DNA recognition domains of Sp1 and Sp3 are highly related such that Sp1 and Sp3 can bind to Sp1-binding sites [31], the ChIP assay previously showed that the GC motifs in the human *MCAK* proximal core promoter appeared to specifically bind only with Sp1 [23]. Consequently, these results demonstrate that p53-mediated repression of the *MCAK* core promoter activity was attributable to p53-dependent inhibition of Sp1-binding to the *MCAK* core promoter via down-regulation of the cellular Sp1 level by p53.

**Fig 5:**
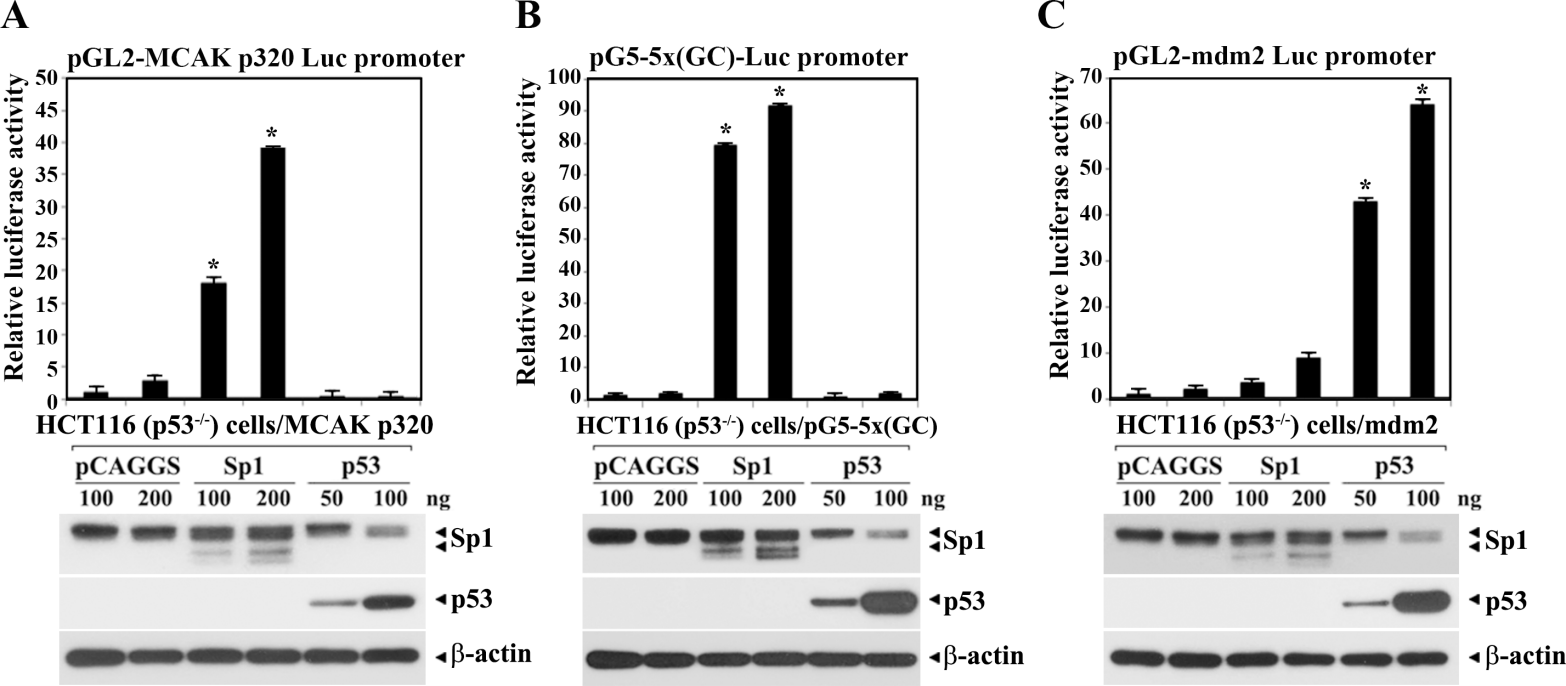
**Differential effect of Sp1 and p53 on the *MCAK* core promoter (pGL2-320-Luc) activity (A), the pG5-5×(GC)-Luc activity (B), and pGL2-mdm2 promoter (pGL-mdm2-Luc) activity (C).** One hundred nanograms of individual promoter-reporter constructs (pGL2-320-Luc, pG5-5×(GC)-Luc, and pGL2-mdm2-Luc) were cotransfected with pCAGGS-Sp1, pCAGGS-wt-p53, or pCAGGS control empty vector into HCT116 (p53^-/-^) cells, and then the luciferase activity was determined. Each value is expressed as the mean ± SD (n = 3). *Statistical significance was defined as *p*<0.05 compared to the control (100 ng of pCAGGS). To analyze the expression level of MCAK, Sp1, and β-actin proteins in the cells after transient transfection, western blot analyses were performed as described in Materials and methods. A representative study is shown and two additional experiments yielded similar results.

To confirm the roles of Sp1 and p53 in regulation of the *MCAK* core promoter activity, either pCMV-Noe-Sp1 or pCMV-Neo-wt-p53 was cotransfected with pG5-5x(GC)-Luc, pGL2-mdm2-Luc or pGL2-320-Luc into HCT116 (p53^−/−^) cells. After incubation for 48 h, the effects of Sp1 and p53 expressions on individual promoter-reporter constructs were measured. As shown in Fig 5A, the luciferase activity of pGL2-320-Luc was significantly and dose-dependently enhanced in the cells following transfection with pCMV-Neo-Sp1 (100−200 ng); however, the promoter activity was completely abrogated in the cells transfected with pCMV-Neo-wt-p53 (50−100 ng). While the activity of the pG5-5×(GC)-Luc construct containing five tandem GC-motifs showed a 38- to 45-fold increase following transfection with pCMV-Neo-Sp1, the activity of the pGL2-mdm2-Luc construct containing p53-RE was enhanced 23- to 30-fold in the presence of wt-p53 (Fig 5B and 5C). Under these conditions, western blot analysis showed successful expression of Sp1 and wt-p53 proteins in HCT116 (p53^−/−^) cells after transfection with individual expression plasmids. Additionally, the level of Sp1 protein markedly decreased when ectopically expressed wt-p53 was accumulated in HCT116 (p53^−/−^) cells. These data indicate that the expressed wt-p53 as well as Sp1 following transfection into HCT116 (p53^−/−^) cells was functional, and support the notion that p53-mediated repression of the *MCAK* core promoter activity was indirectly exerted by p53-dependent down-regulation of Sp1 level.

### Involvement of down-regulation of Sp1 levels in p53-mediated repression in the MCAK promoter activity

**Fig 6:**
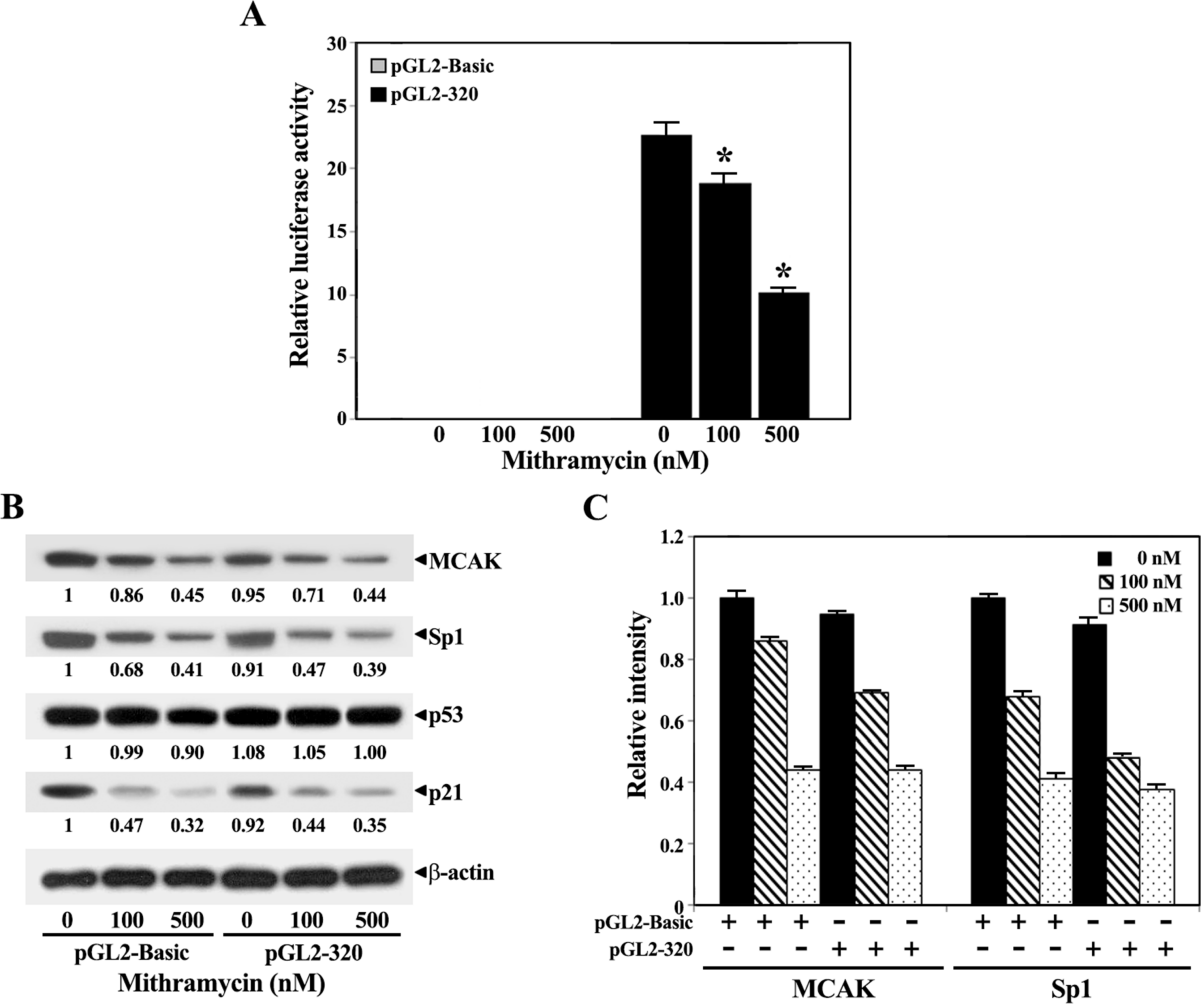
**Effect of mithramycin A on the Sp1-dependent activation of *MCAK* promoter activity of the proximal core *MCAK* promoter pGL2-320 (A) and the endogenous levels of MCAK, Sp1, p53, and β-actin (B), and the arbitrary densitometric units of the individual proteins normalized to those of β-actin (C) in HEK-293 cells.** HEK 293 cells were transiently transfected with either 0.2 μg of the promoter-reporter construct pGL2-320 (-266/+54) or equivalent amount of the control vector pGL2-basic. After transfection for 24 h, the cells were treated with various concentrations of mithramycin A (0, 100, and 500 nM). The luciferase activity was measured 48 h after transfection and normalized for the amount of protein. The mean ± SD of three independent experiments performed in duplicate is shown. *Statistical significance was defined as *p*<0.05 compared to the control. Under the same condition, equivalent amounts of cell lysates were analyzed for the endogenous levels of MCAK, Sp1, p53, p21^WAF1/Cip1^, and β-actin by western blotting. A representative study is shown and two additional experiments yielded similar results.

To verify the contribution of Sp1 to MCAK gene expression, we investigated the effect of mithramycin A on the Sp1-dependent activation of pGL2-320-Luc, because mithramycin A was previously reported to not only down-regulate Sp1 at the transcriptional level but also inhibit sp1 binding to the Sp1 target genes including the p21^WAF1/Cip1^, PUMA, and BAK genes [32, 33]. When HEK-293 cells bearing pGL2-320-Luc were treated with mithramycin A at various concentrations (0, 100, or 500 nM) for 48 h, the luciferase activity decreased in a dose-dependent manner (Fig 6A). Under these conditions, although the p53 level remained relatively constant in the cells following mithramycin A treatment, the levels of Sp1 was markedly reduced (Fig 6B and 6C). In accordance with the decrease in the Sp1 level, the MCAK level was also markedly reduced by mithramycin. Additionally, mithramycin could diminish the p21^WAF1/Cip1^ level, consistent with a previous report demonstrating mithramycin-mediated inhibition of Sp1 binding to the p21^WAF1/Cip1^ promoter [33].

**Fig 7:**
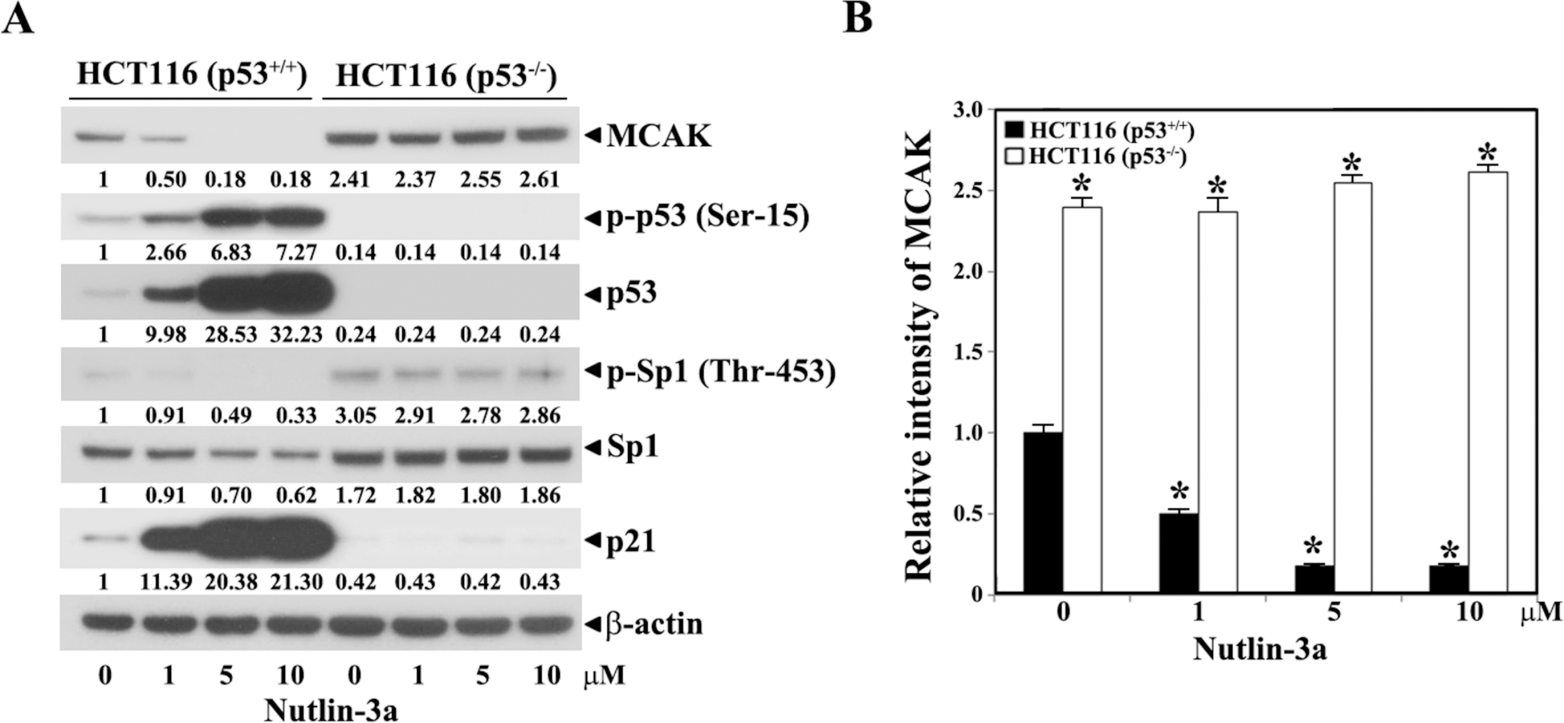
Effect of nutlin-3a on the endogenous levels of MCAK, Sp1, p53, p21^WAF1/Cip1^, and β-actin (B) in HCT116 (p53^+/+^) and HCT116 (p53^−/−^) cells. **The cells were treated with various concentrations of nutlin-3a (1, 5, and 10 μM).** After treatment for 48 h, the cells were harvested and cell lysates were prepared. Equivalent amounts of cell lysates were analyzed for endogenous levels of MCAK, Sp1, p53, p21^WAF1/Cip1^, and β-actin by western blotting. A representative study is shown and two additional experiments yielded similar results. The mean ± SD of three independent experiments performed in duplicate is shown. *Statistical significance was defined as *p*<0.05 compared to the control. The arbitrary densitometric units obtained for the MCAK protein were normalized to those of β-actin in HCT116 (p53^+/+^) and HCT116 (p53^−/−^) cells.

Sequentially, we further compared the contribution of down-regulation of Sp1 level to p53-mediated repression of the *MCAK* core promoter activity in HCT-116 (p53^+/+^) and HCT-116 (p53^−/−^), using nutlin-3a that blocks the p53-mdm2 interaction to stabilize and activate p53 and thus induce the expression of the p21^WAF1/Cip1^ gene [34, 35]. As shown in Fig 7A, HCT116 (p53^+/+^) cells treated with nutlin-3a (1−10 μM) exhibited a concomitant accumulation of p53 and p21^WAF1/Cip1^ proteins. In accordance with the nutlin-3a-induced enhancement of p53 level in HCT116 (p53^+/+^) cells, the Sp1 and MCAK levels were significantly reduced. However, these nutlin-3a-induced alterations including the decrease in Sp1 and MCAK levels were not detected in HCT116 (p53^−/−^) cells. Since the human Sp1 gene was reported to be positively regulated by Sp1 protein through its binding to multiple GC-motifs located within the proximal promoter region [36], the p53-dependent decrease in the cellular Sp1 level was caused by attenuating Sp1-dependent activation of the Sp1 promoter via protein-protein interaction between p53 and Sp1.

These previous and current results indicate that p53-mediated repression of the Sp1-dependent activation of the *MCAK* promoter is associated with down-regulation of the cellular Sp1 level by p53; however, a concurrent inhibition of Sp1 activity by p53 via protein-protein interaction cannot be excluded.

### Effect of p53 mutants on the MCAK core promoter activity

Although current results shows that the human MCAK gene is a target for p53-mediated repression, the mechanism for tumor-associated elevation of MCAK levels still remains obscure. As one of the most frequent genetic alterations detected in human cancer cells (>50%), several missense mutation patterns have been found in p53 [37, 38]. These tumor-associated p53 mutations reside mainly in the DNA-binding domain, and impair its ability as a transcription factor to regulate p53-responsive genes. As oncogenic properties of the mutant p53, loss of tumor suppression activity causing accelerated cell proliferation and resistance to chemotherapeutic drugs have been implicated [37]. Given that several studies have shown elevated expression of the MCAK gene in human cancer cells [17–20], it is likely that tumor-derived p53 mutants are unable to repress the *MCAK* promoter activity as efficiently as wt-p53.

**Fig 8:**
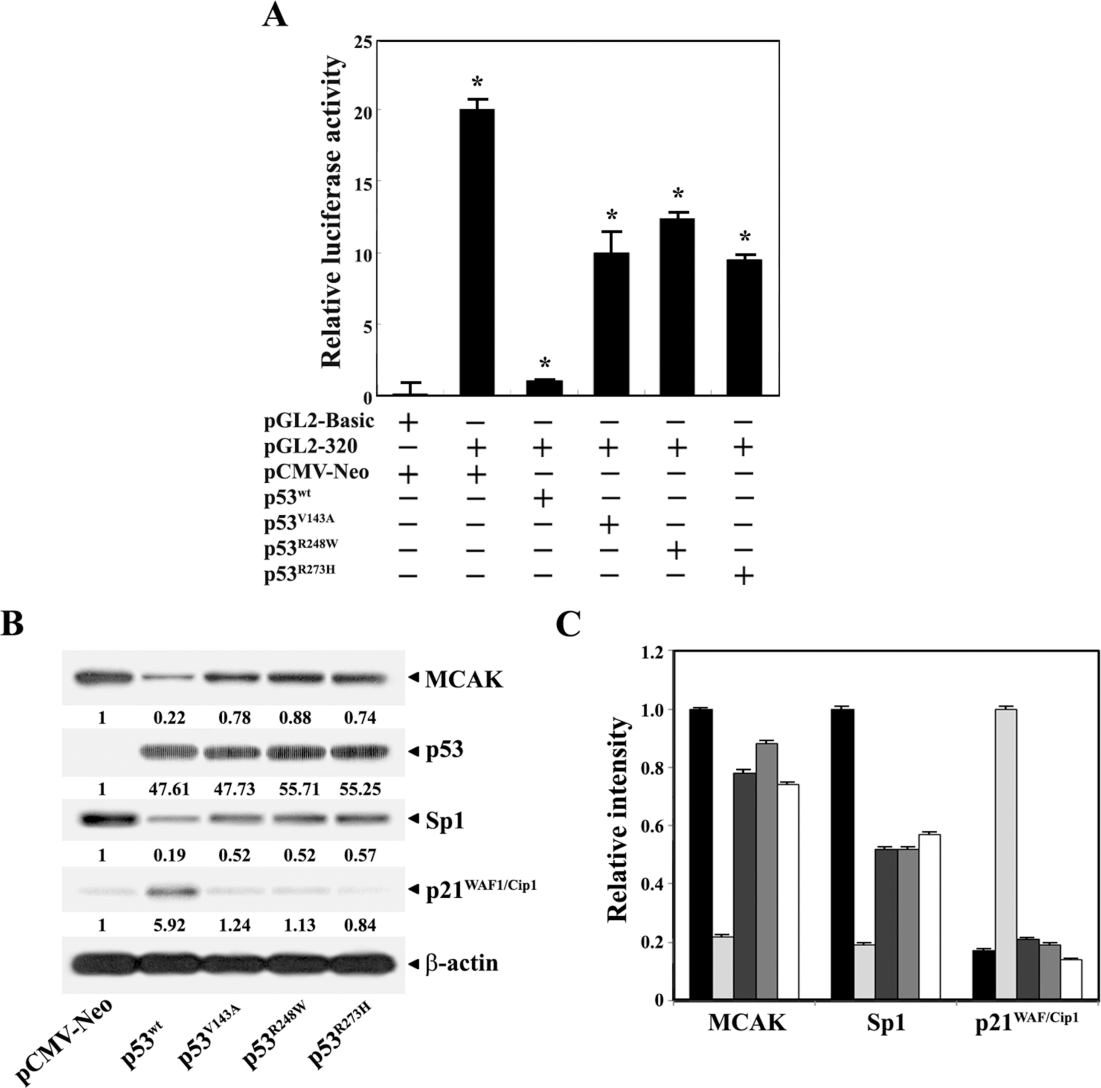
**Differential effect of wild-type p53 or tumor-derived p53 mutants on the MCAK core promoter (pGL2-320-Luc) activity (A), and the endogenous levels of MCAK, p53, Sp1, p21**^**WAF1/Cip1**^**, and β-actin (B), and the arbitrary densitometric units of the individual proteins normalized to those of β-actin (C) in HCT116 (p53**^−**/**−^**) cells.** The promoter-reporter construct pGL2-320 was cotransfected into HCT116 (p53^−/−^) cells with empty-vector (pCMV-Neo), or each expression construct of wt-p53 (pCMV-Neo/p53^wt^), mutant p53^V143A^ (pCMV-Neo/p53^V143A^), mutant p53^R248W^ (pCMV-Neo/p53^R248W^), and mutant p53^R273H^ (pCMV-Neo/p53 ^R273H^), and then luciferase activity was determined. Each value is expressed as the mean ± SD (n = 3). *Statistical significance was defined as *p*<0.05 compared to the control. Under these conditions, equivalent amounts of cell lysates were analyzed for the endogenous levels of individual proteins by western blotting. A representative study is shown and two additional experiments yielded similar results.

To examine whether tumor-derived p53 mutants are able to down-regulate the *MCAK* core promoter activity as efficiently as wt-p53, pGL2-320-Luc was cotransfected with individual expression vectors for wt p53, p53^V143A^, p53^R248W^, and p53^R273H^ into HCT116 (p53^−/−^) cells and then the luciferase activity of pGL2-320-Luc was measured. As shown in Fig 8A, the luciferase activity of pGL2-320-Luc was reduced by ~90% in HCT116 (p53^−/−^) cells following cotransfection with pCMV-Neo-wt p53. However, cotransfection of pGL2-320-Luc with each p53 mutant expression vector for p53^V143A^, p53^R248W^, or p53^R273H^, resulted in only ~50%, ~40%, and ~48% reduction of the luciferase activity, respectively. These results demonstrate that mutant p53^V143A^, mutant p53^R248W^, or mutant p53^R273H^ repressed the *MCAK* promoter activity to a much lower extent as compared to wt-p53. Under these conditions, western blot analysis also showed that p53 mutants failed to repress the endogenous MCAK level as efficiently as wt-p53 in HCT116 (p53^−/−^) cells (Fig 8B and 8C). While the ectopic expression levels of wt-p53 and individual p53 mutants in HCT116 (p53^−/−^) cells were similar in their quantities, the endogenous MCAK level in HCT116 (p53^−/−^) cells was reduced to ~22% in the presence of wt-p53, but the levels of 78%, 88%, and 74% were maintained in the presence of mutants p53^V143A^, p53^R248W^, and p53^R273H^, respectively. At the same time, the endogenous Sp1 level in HCT116 (p53^−/−^) cells appeared to decrease to ~19% in the presence of wt-p53, whereas it decreased to 52%−57% in the presence of the p53 mutants. Additionally, only wt-p53, in contrast to the p53 mutants, was able to enhance the expression level of p21^WAF1/Cip1^ in HCT116 (p53^−/−^) cells, confirming that the DNA-binding domain of p53 mutants was not functional. These results demonstrate that wt-p53, but none of the p53 mutants, could efficiently repress the *MCAK* core promoter activity via down-regulating the expression level of Sp1 that is crucial for the *MCAK* promoter activity. Consequently, these results suggest that the elevation of MCAK levels in tumors is due to the functional impairment of p53 resulting from tumor-associated mutations, which have frequently been observed in many types of tumors [37, 38].

**Fig 9:**
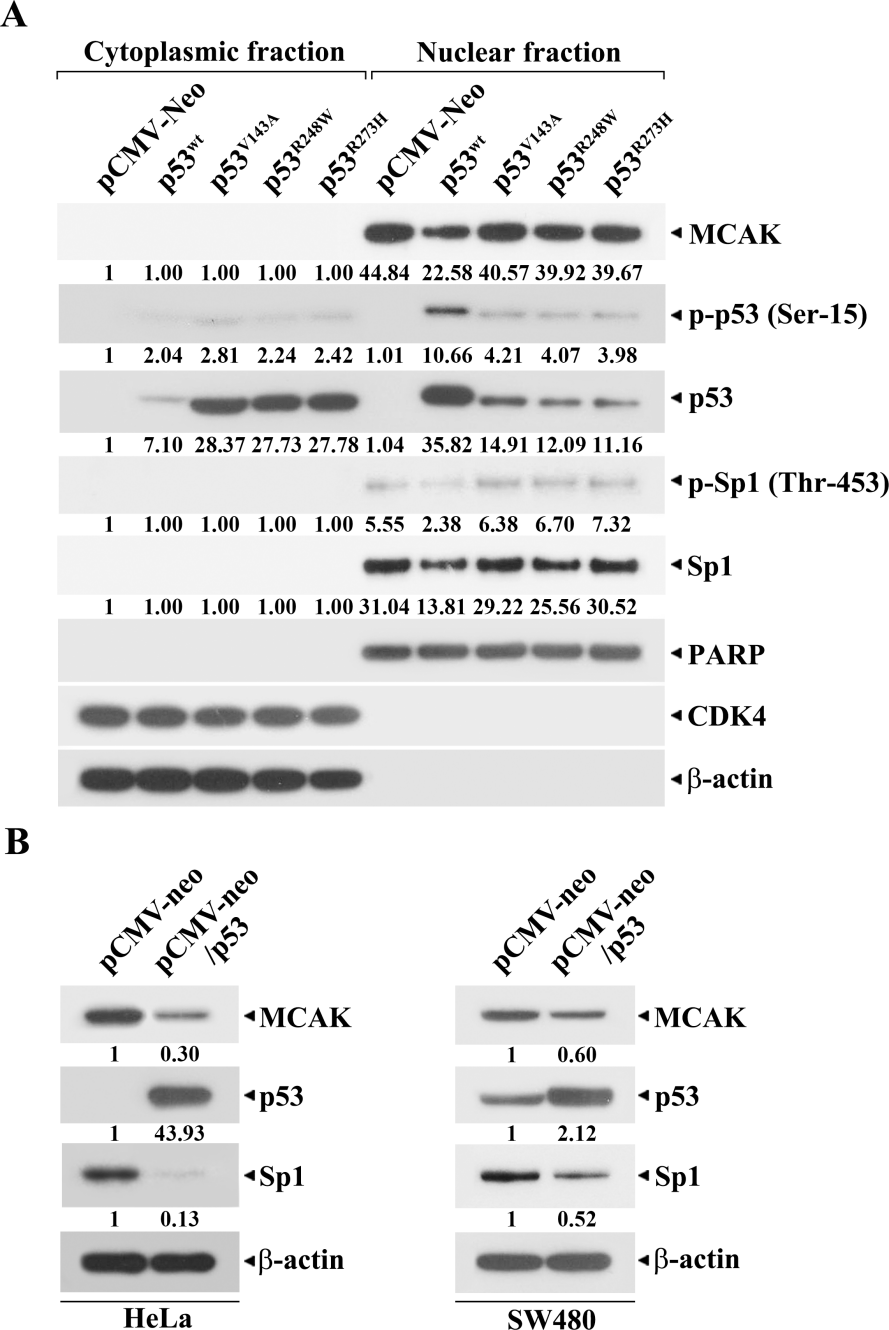
**Cytoplasmic and nuclear localizations of Sp1, wt-p53, and p53 mutants (p53**^**V143A**^**, p53**^**R248W**^**, and p53**^**R273H**^**) in HCT116 cells (A), and ectopic p53 expression-mediated down-regulation of MCAK in HeLa and SW480 (B).** After HCT116 (p53^-/-^) cells were transfected with pCMV-Neo, pCMV-wt-p53, pCMV-p53^V143A^, pCMV-p53^R248W^, and pCMV-p53^R273H^, the cells were fractionated into cytosolic and nuclear fractions. Western blot analyses were performed to assess the subcellular localization of MCAK, p-p53 (Ser-15), p53, p-Sp1 (Thr-453), Sp1, PARP, CDK4, and β-actin. CDK4 and β-actin were used as cytoplasmic markers, and PARP was used as the nuclear marker. Both HeLa and SW480 cells transfected with pCMV-Neo and pCMV-wt-p53, respectively, were subjected to western blot analysis of MCAK, p53, Sp1, and β-actin. A representative experiment is shown, and two additional experiments yielded similar results.

Previously, it has been shown that p53 mutations mainly reside in the DNA-binding domain [39, 40]. Mutations in the DNA-binding domain of p53 cause distinct functional alterations, including impairment of the interaction of p53 with its responsive elements of target genes, enhanced degradation of p53, or cytoplasmic sequestration of p53, leading to either loss of function or gain of function in transcriptional activity of p53 [40]. In order to examine whether p53 mutations (p53^V143A^, p53^R248W^, and p53^R273H^), alterations of which reside in the DNA-binding domain [37, 38], could alter their subcellular localization to render p53 unable to repress target genes, we investigated the levels of wt-p53 and p53 mutants localized in the cytosolic and nuclear fractions along with their phosphorylation status in HCT116. As shown in Fig 9A, when the integrity of the individual fractions was analyzed by western blot analysis with PARP as a nuclear marker, and cdk4 and β-actin as cytosolic markers, the individual proteins appeared to be located in their reference fractions. At the same time, both MCAK and Sp1 proteins were mainly detected in the nuclear fraction. Although ~83.4% of wt-p53 was detected in the nuclear fraction, the ratios of p53^V143A^, p53^R248W^, and p53^R273H^ mutants in the nuclear fraction were ~34.5%, ~30.4%, and ~28.7%, respectively, indicating that nuclear localization of p53 could be impaired by mutation of the DNA-binding domain of p53. Under these conditions, the p53 mutations appeared to restore the wt-p53-dependent decrease in the cellular Sp1 level; however, the p53 mutations did not influence the nuclear localization of Sp1 or its phosphorylation at Thr-453. Consequently, these results indicate that p53 mutations (p53^V143A^, p53^R248W^, and p53^R273H^) could inhibit the nuclear translocation of p53, and thus reduce both p53-interactions with Sp1 and p53-mediated down-regulation of the Sp1 level in the nucleus.

On the other hand, ectopic expression of wt-p53 in HeLa and SW480 cells, both of which are known to carry compromised p53 [41, 42], down-regulated the MCAK level (Fig 9B). These results suggest that wt-p53-mediated negative regulation of the MCAK expression level observed in human colorectal adenocarcinoma HCT116 cells can be extended to human cervical cancer HeLa cells and human colon adenocarcinoma SW480 cells.

In conclusion, this is the first report to demonstrate that the human *MCAK* core promoter (−266 to +54, relative to the transcription start site) is repressed by wt-p53, whereas tumor-derived p53 mutants, such as p53^V143A^, p53^R248W^, and p53^R273H^, cannot repress the *MCAK* promoter as efficiently as wt-p53. The tumor suppressor protein p53-mediated repression of the *MCAK* promoter was not directly mediated by p53-binding to p53-REs in the promoter, but indirectly by interfering with the role of transcriptional activator Sp1 which could bind to the GC-motifs within the core positive regulatory region of the *MCAK* promoter. The p53-mediated repression of the Sp1-dependent activation of the *MCAK* promoter is caused by down-regulation of the Sp1 level by p53 at the transcriptional level. Tumor-associated elevation of MCAK levels appears to be mainly due to malfunction of tumor-derived p53 mutants, which failed to repress the *MCAK* promoter as efficiently as did wt-p53.
